# In Vitro Nuclear Assembly Using Fractionated Xenopus Egg Extracts

**DOI:** 10.3791/908

**Published:** 2008-09-02

**Authors:** Marie Cross, Maureen Powers

**Affiliations:** Department of Cell Biology, Emory University

## Abstract

Nuclear membrane assembly is an essential step in the cell division cycle; this process can be replicated in the test tube by combining Xenopus sperm chromatin, cytosol, and light membrane fractions. Complete nuclei are formed, including nuclear membranes with pore complexes, and these reconstituted nuclei are capable of normal nuclear processes.

**Figure:**
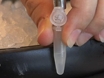


## Protocol

The complete text protocol for this experimental approach is available in Current Protocols in Cellular Biology.

